# Impotenz und Hexenglauben: Ein medizinischer Traktat des Ulmer Stadtarztes Wolfgang Reichart (1486–1547)

**DOI:** 10.1007/s00120-020-01136-6

**Published:** 2020-02-07

**Authors:** Frank Ursin, Giovanni Rubeis, Florian Steger

**Affiliations:** 1 Institut für Geschichte, Theorie und Ethik der Medizin, Universität Ulm, Parkstraße 11, 89073 Ulm, Deutschland; 2 Institut für Geschichte und Ethik der Medizin, Ruprecht-Karls-Universität Heidelberg, Im Neuenheimer Feld 327, 69120 Heidelberg, Deutschland

**Keywords:** Renaissance, Pathophysiologie, Therapie, Erektile Dysfunktion, Medizingeschichte, Kasuistik, Renaissance, Pathophysiology, Therapy, Erectile dysfunction, History of medicine, Casuistry

## Abstract

**Hintergrund:**

Der Hexenglaube beeinflusste im 16. Jahrhundert das medizinische Denken und Handeln. Der Ulmer Stadtarzt Wolfgang Reichart (1486–1547) hat in einem bisher unbekannten Traktat die Impotenz eines Patienten mit medizinischen Konzepten rational erklärt.

**Material und Methode:**

Der Traktat wurde transkribiert, übersetzt und hinsichtlich seiner Quellen, Struktur und des Inhalts analysiert. Die Ergebnisse wurden mit Johann Weyers (1515–1588) Konzepten verglichen.

**Ergebnisse:**

Reichart erklärt die Impotenz seines Patienten als erworbene Erkrankung, an der Dämonen beteiligt waren. Da Dämonen nur auf natürliche Weise auf den menschlichen Körper wirken, sei die Krankheit auf natürlichem Weg heilbar. Grundlage der Therapie ist ein mittelalterliches pathophysiologisches Konzept, das antike Elemente kombinierte.

**Schlussfolgerungen:**

Reicharts Therapie unterscheidet sich von derjenigen zeitgenössischer Ärzte, weil er den Patienten selbst behandelt und nicht an einen Theologen überweist. Im Gegensatz zu Weyer bietet er ein detailliertes pathophysiologisches Konzept zur medizinischen Erklärung der Impotenz.

## Abstract (en)

**Background:**

The belief in witchcraft influenced medical thinking and action in the 16th century. In a hitherto unknown treatise on impotence, the Ulmian town physician Wolfgang Reichart (1486–1547) has rationally explained it by using medical concepts.

**Materials and methods:**

The treatise was transcribed, translated, and analyzed in terms of its sources, structure, and content. The results were compared with the concept of Johann Weyer (1515–1588).

**Results:**

Reichart explains his patient’s impotence as an acquired disease involving demons. Since demons act only naturally on the human body, the disease is naturally curable. The basis of the therapy is a medieval pathophysiological concept that combined ancient elements.

**Conclusions:**

Reichart’s therapy differs from that of contemporary physicians because he treats the patient himself and does not send him to a theologian. Unlike Weyer, he offers a detailed pathophysiological concept in order to explain impotence medically.

## Hintergrund

Der Hexenglaube beeinflusste im 16. Jahrhundert das medizinische Denken und Handeln. Der Arzt Johann Weyer (1515–1588) hat sich mit seinem einflussreichen Buch *De praestigiis daemonum* (1563) als einer der Ersten dafür eingesetzt, Hexen für plötzliche Erkrankungen nicht zur Verantwortung zu ziehen [29, 30]. Die Opfer von Hexerei wurden in den folgenden Jahren vermehrt von einem Arzt behandelt anstatt an einen Priester überwiesen. Bis dahin kursierte seit dem späten Mittelalter in medizinischen Fachschriften die Ansicht, dass Hexen für plötzliche Fälle von männlicher Impotenz verantwortlich sein können [20, 26]. Maßgeblich wurde diese Vorstellung geprägt vom sog. Hexenhammer (*Malleus maleficarum*, 1486/7, [2, 10]).

Ein neugefundener medizinischer Traktat liefert nun einen Beitrag zur Entwicklung des Hexenglaubens innerhalb der frühneuzeitlichen Medizin. Der Traktat *Conclusiones super quodam eunucho infrigidato* („Schlussfolgerungen über einen gewissen erkalteten Eunuchen“) wurde von den Autoren in der Briefsammlung des Ulmer Stadtarztes Wolfgang Reichart (1486–um 1547, Abb. 1 und 2) entdeckt [15]. Dieser hat eine Generation vor Weyer gelebt. Das Besondere daran ist, dass Reichart im Gegensatz zu Weyer medizinisch-rationale und nicht theologische Konzepte auf übernatürliche Ursachen anwandte. Damit stellt er das seltene Beispiel eines frühneuzeitlichen Arztes dar, der seinen Patienten hinsichtlich der Impotenz nicht für einen Exorzismus zu einem Priester sandte, sondern mit den medizinischen Mitteln der damaligen Zeit in Behandlung nahm.

**Figure 1:**
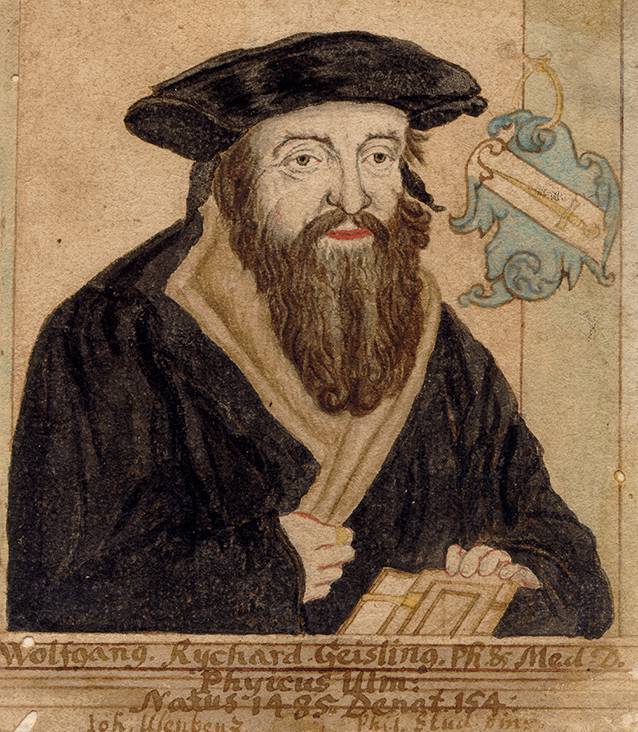

**Figure 2:**
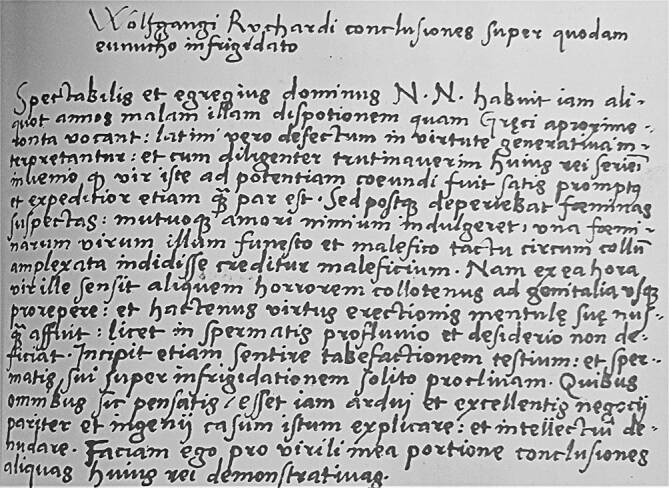

Das neue Fundstück lädt dazu ein, Reicharts pathophysiologische und therapeutische Erklärungen mit denen Johann Weyers zu vergleichen. Dadurch können wir zeigen, dass Reichart medizinische Rationalität zur Erklärung übernatürlicher Ursachen benutzte und dies über Weyers Ätiologie der Impotenz hinausgeht.

Reicharts ungenannter Patient berichtet, dass eine Frau durch das Würgen seines Halses einen Schauder ausgelöst habe, der vom Hals bis zu den Genitalien hinuntergereicht habe. Daraufhin habe der Patient die *Potentia coeundi* verloren, d. h. das Vermögen Geschlechtsverkehr auszuüben. Der Patient konnte noch ejakulieren, verlor aber die Erektionsfähigkeit.

Wolfgang Reichart, 1486 in Geislingen geboren, besuchte die Lateinschule im Kloster Blaubeuren und erhielt in Tübingen den Baccalaureus (1502) und Magistergrad (1509). 1512 wurde er in Freiburg promoviert und ein Jahr später Stadtarzt von Ulm [15]. Ab 1508 war er medizinischer Gehilfe des Ulmer Stadtarztes Johannes Stocker (1453–1513). Reichart war ein dem Humanismus zugewandter Arzt und sympathisierte in den 1520er-Jahren mit der Reformation. 1523 sandte er zwei Rezepte an Martin Luther (1483–1546), womit dieser sein aufgrund geistiger Überanstrengung „zu trockenes Gehirn“ behandeln sollte [24]. Bis zu seinem Tod um 1547 blieb Reichart Stadtarzt in Ulm.

## Material und Methoden

### Quellen

Die frühneuzeitliche Abschrift der 589 Texte Reicharts lagert in der Staats- und Universitätsbibliothek Hamburg [12, 24]. Der größte Teil der Sammlung beinhaltet die Korrespondenz mit seinem Sohn Zeno Reichart (1507–1543), Geistlichen und Ärzten [17]. Etwa 60 Briefe thematisieren medizinische Themen und stellen Konsile dar [23]. Bisher ist ein Traktat über die Besessenheit eines Knaben bekannt, die Reichart als Melancholie diagnostizierte (Sup. Ep. 4° 49 Nr. V; [16]). Jetzt tritt der neue Traktat über die Pathophysiologie und Therapie der Impotenz hinzu (Nr. CCCXLVI; [24]). Dieser Text ist nicht datiert, dürfte aber aus den 1530er-Jahren stammen, weil inhaltliche und sprachliche Ähnlichkeiten sowie das methodische Vorgehen mit dem bereits bekannten Melancholie-Traktat von 1534 übereinstimmen.

### Methodisches Vorgehen

Wir haben den lateinischen Traktat transkribiert und übersetzt. Anschließend haben wir ihn analysiert in Hinsicht auf seine Quellen, Struktur und Inhalt. Die inhaltliche Kontextualisierung erfolgte anhand der einschlägigen Literatur zur Medizin- und Wissenschaftsgeschichte sowie zu medizinischen und theologischen Konzepten der Impotenz im 16. Jahrhundert [5, 19–22, 25–27, 30]. Reicharts pathophysiologisches Konzept wurde abschließend mit dem Weyers verglichen [29, 30].

## Ergebnisse und Diskussion

### Struktur und Terminologie

Der Traktat ist in ein Vorwort, vier Schlussfolgerungen (*conclusiones*) und einen therapeutischen Teil gegliedert. Reichart beginnt mit der Fallvorstellung eines namenlosen *Aproximeroūnta*-Patienten (einer, der unter der Untätigkeit seines Gliedes leidet). Diese griechische Bezeichnung setzt sich aus *apraxía* (Untätigkeit) und *méros* (Teil) zusammen, was mittelalterliche Autoren als *genitalium resolutio* (Peniserschlaffung) oder *erigendi et generandi impotentia* (Erektions- und Zeugungsunfähigkeit) lateinisch fassten [18]. In antiken medizinischen Fachschriften ist der griechische Terminus nicht belegt [14].

Der Begriff *eunuchus* lässt sich auf das Altgriechische *eunoūchos* zurückführen, was in den hippokratischen Schriften „Über Luft, Wasser und Orte“ (Kap. 22) und „Über die Zeugung“ (Kap. 2) einen zeugungsunfähigen Mann bezeichnet [7, 8]. Daneben verwendet Reichart folgende Begriffe: einmal *ablatio potentiae coeundi* (der Verlust der Fähigkeit zum Beischlaf), einmal *impotentia erectionis*, und 5‑mal *impotentia* in einer allgemeinen Bedeutung.

Der Impotenz-Begriff war historisch gesehen lange Zeit verbreitet, wurde aber im Jahr 1992 zugunsten des Begriffs der erektilen Dysfunktion präzisiert [9]. Weil wir uns der Grenzen retrospektiver Diagnosen bewusst sind [13], verwenden wir im Folgenden weiterhin den Quellenbegriff „Impotenz“, auch wenn es sich um eine erektile Dysfunktion gehandelt haben könnte.

#### Erste Schlussfolgerung: erworbene Erkrankung

In der ersten Schlussfolgerung wird festgestellt, dass die Affektion auf ein bestimmtes Ereignis zurückgeführt werden kann und daher nicht angeboren war. Eine Frau habe den Patienten am Hals gewürgt, woraufhin ein *defectus (potentiae) coeundi* (Fehlen der Fähigkeit zum Beischlaf) eingetreten sei. Da die Werke der Natur Bewegung sind, sei die *ablatio potentiae coeundi* ein permanenter Verlust an Bewegung. Bewegung vollziehe sich immer prozesshaft und nie plötzlich. Reichart schlussfolgert, dass die Frau einen Zauber (*maleficium*) bewirkt habe, der auf die Natur gewirkt habe.

Reichart greift für seinen Erklärungsansatz auf die aristotelische Bewegungstheorie zurück (Physik III 1; [28]), wonach Bewegung als Prozess zu verstehen ist, der eine Ursache hat. Demnach kann das Ereignis nicht spontan geschehen sein, sondern muss natürliche Ursachen haben. Reichart argumentiert, dass auch ein Zauber nur ein Glied in der Kette natürlicher Ursachen ist.

#### Zweite Schlussfolgerung: Bestimmung des Zeitpunkts

Reicharts Prämisse ist, dass sich hinter den Ohren Venen befinden, die bis zu den Genitalien hinabführen. Wenn diese Venen zur Ader gelassen werden, folge Sterilität (*sterilitas*). Diese Venen können durch eine bösartige Berührung (*tactus maleficus*) beschmutzt werden, wenn Gott es erlaube und der Teufel beteiligt ist, wodurch die Zeugungskraft (*virtus generativa*) zerstört wird.

Reichart greift direkt auf pathophysiologische Vorstellungen der Antike zurück, was durch einen Textvergleich bestätigt wird. In der hippokratischen Schrift „Über die Zeugung“ (Kap. 1–2) wird die Ansicht vertreten, dass das Sperma im Gehirn gebildet und durch die Halsvenen über das Rückenmark zu den Hoden transportiert werde [8]. Zwei mögliche Ätiologien für die Entstehung eines Eunuchen werden genannt: Erstens könne der Mann nach einem Schnitt hinter den Ohren zwar noch Geschlechtsverkehr haben und ejakulieren, das Ejakulat sei aber wenig, schwach und steril (*olígos, asthenés, ágonos*). Zweitens verliere der Penis seine Erektionsfähigkeit, wenn die Passage von den Hoden zum Penis zerschnitten werde. Reichart hat also unter Rückgriff auf etablierte pathophysiologische Konzepte erstens darauf hingewiesen, dass die Impotenz seines Patienten auf natürlichem Weg verursacht worden sein kann. Zweitens hat er den Zeitpunkt für die Entstehung der Erkrankung bestimmt.

#### Dritte Schlussfolgerung: pathophysiologische Theorie

Reichart präsentiert eine Theorie für die *potentia coeundi*, die auf drei Bedingungen fußt: Es braucht den Saft (*humor*) aus dem Gehirn für das Sperma, Luft (*spiritus ventosus*) aus dem Herzen als Material der Erektion, und Wärme (*calor desiderii*) aus der Leber für die Libido. Weil dem Patienten weder Sperma noch Libido fehle, sondern allein die Erektion des Gliedes (*sola membri erectio*), muss es eine verborgene Blockade der Passagen zwischen dem Herzen und dem Genitale geben, sodass die Luft (*spiritus ventosus*) das Glied nicht aufrichten kann. Das Glied selbst sei nicht zu behandeln, schließt Reichart, weil es gesund sei und seine Funktion verrichte. Therapeutisch seien demnach die Wege vom Herzen zum Genitale zu versorgen.

Elemente dieser Theorie wurden in der Antike grundgelegt: In der hippokratischen Schrift „Über die Zeugung“ sind bereits *phlégma* (*humor*) und *thérme* (*calor*) genannt (Kap. 1–2; [8]). Galen (129–210 n. Chr.) vertrat in der Schrift „Vom Gebrauch der Körperteile“ (S. 183) die Ansicht, dass die *Corpora cavernosa* in Analogie einer mechanischen Pneumatik mit Luft (*pneúma,* *sprititus*) aufgepumpt würden [6]. Die drei Bedingungen erscheinen gemeinsam erst im Mittelalter in der Schrift *De Coitu* bei Constantinus Africanus (gestorben vor 1099, Kap. 1–2; [4]).

#### Vierte Schlussfolgerung: Degeneration durch Inaktivität

Avicenna (ca. 980–1037) ist der einzige Autor, auf den Reichart namentlich verweist. Aus einem Brief ist bekannt, dass Reichart seinem Sohn Zeno den *Canon* Avicennas zusammen mit dem Kommentar des Jacques Despars (um 1380–1458) für das Studium der Medizin empfiehlt [16]. Avicenna wird für das Prinzip zitiert, dass Körperteile verkümmern, wenn sie nicht benutzt werden [1]. Daher verwundere es nicht, dass Penis und Hoden des Patienten degeneriert seien. Der Grund dafür sei, dass das Genitale zunehmend erkaltet sei, weil mehrere Jahre keine Bewegung, Reibung und damit Wärme hervorgerufen wurde. Hintergrund für die Beobachtung und Erklärung könnte eine tatsächliche Atrophie des Genitale gewesen sein.

#### Therapeutischer Teil

Gemäß den Theologen sei eine durch Verhexung (*maleficium*) hervorgerufene Impotenz unheilbar. Da Reichart geschlussfolgert hatte, dass Dämonen als Verursacher in Frage kommen können, kann er zwar keine Heilung versprechen. Weil Dämonen aber auf natürliche Weise die identifizierte Blockade errichtet haben, könne diese auch auf natürliche Weise beseitigt werden.

Als therapeutisches Ziel formuliert Reichart, dass das Herz den *Spiritus ventosus* erzeugen muss und angeregt wird, ihn an die Geschlechtsorgane zu lenken. Dieses Ziel erfüllen Medikamente und Diätetik, verstanden als Nahrungs- und Lebenswandelumstellung (Tab. 1, 2 und 3). Das von Reichart empfohlene Medikament ist ein unspezifisches Gewürz (*condimentum*), das jeden Morgen und Abend eingenommen werden soll. Parallel dazu sollen Penis und Hoden mit einer nicht näher beschriebenen Salbe bestrichen werden. Außerdem solle Gott angerufen werden, denn erstens gefalle es ihm, und zweitens stärke dies die Seele des Kranken.

**Table 1:** 

Struktur	Inhalt	
Vorwort	Kasuistik	Ein namenloser *Aproximeroūnta*-Patient (einer, der unter der Untätigkeit seines Gliedes leidet) wird vorgestellt
	Methodisches Vorgehen	Deduktiv-deskriptiv (*conclusiones demonstrativas*)
1. Schlussfolgerung	Es handelt sich um eine erworbene Erkrankung, deren Ursachen natürlich sind	
2. Schlussfolgerung	Bestimmung des Zeitpunkts: Die Berührung der Frau kann zusammen mit dem Wirken von Dämonen die Ursache sein	
3. Schlussfolgerung	Pathophysiologische Theorie (drei Bedingungen für die *potentia coeundi*: Sperma aus dem Gehirn, Luft aus dem Herzen und Libido aus der Leber): Es fehlt an Luft	
4. Schlussfolgerung	Durch Inaktivität des Genitale ist es verkümmert	
Therapeutischer Teil	Therapieanweisungen werden genannt, gegliedert nach Diätetik und Medikamenten

**Table 2:** 

Organ	Produkt	Effekt
*Cerebrum* (Gehirn)	*Humor* (Saft)	Sperma für die Zeugung
*Cor* (Herz)	*Spiritus ventosus* (Luft)	Medium der Erektion
*Hepar* (Leber)	*Calor desiderii* (Hitze der Begierde)	Libido für den Verkehr

**Table 3:** 

Therapieansatz	Therapeutika	
Medikamente	Gewürz (*condimentum*), Salbe	
Diätetik	Pflanzliche Nahrungsmittel	Pfeffer, Safran, Ingwer, Zitwerwurzel (*Curcuma zedoaria*), Brunnenkresse (*Nasturtium officinale*), Kubeben-Pfeffer (*Piper cubeba*), schwarze Mandeln, Feigen, Haselnüsse, Rüben, Hülsenfrüchte
	Tierische Nahrungsmittel	Eigelb, frisches Fleisch, Butter, Innereien von Tieren, Gehirne von Vögeln
	Empfohlene Tätigkeiten	Mai-Bäder, tiefer Schlaf, Muße, moderates Essen, Musik, Freude
	Nicht empfohlene Gemütszustände	Unruhe, Sorge

### Vergleich mit zeitgenössischer Ätiologie, Pathophysiologie und Therapie

Reicharts Traktat baut auf dem Hexenglauben der Frühen Neuzeit auf. Im Hexenhammer (1486/7) wurden Hexen für männliche Impotenz und das Verschwinden von Penissen verantwortlich gemacht [10]. Dort wurde geraten, die Hexe zu überreden, die Potenz wieder herzustellen und wenn das nicht helfe, Gewalt anzuwenden. Reicharts therapeutischer Ansatz zielt nicht auf die Hexe als Urheberin, sondern auf den zu behandelnden Patienten. Die Hexe als Urheberin spielt bei Reichart nur insofern eine Rolle, als sie mithilfe von Dämonen eine natürliche Obstruktion der Gefäße zwischen dem Herzen und Genitale des Patienten errichtet hat.

Reichart hat die Ansicht Johann Weyers vorweggenommen, dass Dämonen und nicht eine Hexe Impotenz hervorrufen (*De praestigiis daemonum*, Buch IV, Kapitel XX) [20]. Weyer nennt als weitere Ursachen in nur einem Satz natürliche Prozesse, Unfälle oder die Verabreichung von Drogen. Weder erklärt Weyer diese Ursachen so ausführlich wie Reichart, noch nennt er eine Therapie. Das Ziel seiner Darstellung ist die Bekämpfung von Aberglauben. Vor diesem Hintergrund ist Reicharts Ansatz originell, weil er übernatürliche Ursachen mithilfe detaillierter medizinischer Konzepte auf natürliche Ursachen zurückführt und eine Therapie anbietet. Diese Therapie steht in der Tradition persisch-arabischer Medizin, die durch Constantinus Africanus, Avicenna und Rhazes (865–925) vermittelt wird, wie ein Vergleich der von Reichart empfohlenen Drogen nahelegt [11].

Die Fortschritte im physiologischen Verständnis der Erektion im 15. und 16. Jahrhundert hatten keinen Einfluss auf Reichart. Der medizinische Laie Leonardo da Vinci (1452–1519) hatte beobachtet, dass ein gesteigerter Blutfluss in den Penis für eine Erektion verantwortlich ist [26]. Da er seine Beobachtungen nie veröffentlicht hat, dauerte es über 100 Jahre bis der Chirurg Ambroise Paré (1510–1590) zu dem gleichen Ergebnis kam [26]. Dies hatte keinen Einfluss auf Reichart, der in der Tradition von Constantinus Africanus *calor, spiritus* und *humor* als die drei notwendigen Bedingungen für eine Erektion annahm [3]. Reichart vertrat die antike Vorstellung, wonach die *Corpora cavernosa* bei einer Erektion mit Luft (*spiritus*) gefüllt wurden.

## Zusammenfassung

Der Vergleich von Wolfgang Reicharts Traktat mit pathophysiologischen und therapeutischen Ansätzen zur Impotenz des 16. Jahrhunderts hat drei neue Erkenntnisse ergeben. Die Therapie Reicharts unterscheidet sich von der zeitgenössischer Ärzte, die den Patienten entweder an einen Theologen überwiesen oder eine Hexe mit Gewalt zu einer Heilung des Patienten gezwungen haben. Reichart behandelt seinen Patienten mit Medikamenten und Diätetik, die in der Tradition persisch-arabischer Medizin stehen.

Reichart bietet im Gegensatz zu dem eine Generation später schreibenden Weyer ein detailliertes pathophysiologisches Konzept zur Erklärung der Impotenz heran. Dieses Konzept entspricht dem Konzept von Constantinus Africanus. Eine Hexe spielt bei diesem rationalen Vorgehen im Gegensatz zum Hexenhammer und Weyer nur eine untergeordnete Rolle, weil der Patient im Mittelpunkt steht.

Reicharts Traktat trägt zur Wissenschaftsgeschichte des 16. Jahrhunderts bei. Obwohl er nie im Druck erschien und daher keine Rezeption erfuhr, stellt das methodische Vorgehen ein Dokument des Übergangs von der mittelalterlichen Scholastik hin zu einer empirisch orientierten Medizin dar. Die Gliederung des Traktats in Schlussfolgerungen, die jeweils ausgehend von medizinischen oder theologischen Axiomen rational abgeleitet werden, ist zwar nicht neu. Auch sind die zugrunde gelegten Kenntnisse der menschlichen Anatomie und die pathophysiologischen Vorstellungen aus heutiger Sicht falsch. Aber der Versuch, übernatürliche Vorgänge zwingend auf natürliche Ursachen zurückzuführen, hat sich letztlich durchgesetzt.
